# Barriers and facilitators to telerehabilitation adherence in young and middle-aged stroke survivors: a qualitative COM-B analysis

**DOI:** 10.3389/fneur.2026.1885751

**Published:** 2026-09-28

**Authors:** Yiling Huang, Meilan Su, Yingying Chen, Sufang Hu

**Affiliations:** Department of Rehabilitation, Zhongshan Hospital of Xiamen University, School of Medicine, Xiamen University, Xiamen, China

**Keywords:** adherence, barriers, COM-B model, facilitators, qualitative research, stroke, telerehabilitation, young and middle-aged

## Abstract

**Objective:**

To explore the barriers and facilitators to telerehabilitation adherence among young and middle-aged stroke survivors using the Capability, Opportunity, Motivation–Behavior (COM-B) model.

**Methods:**

A qualitative descriptive study was conducted with 15 stroke survivors aged 18–60 years recruited from a tertiary hospital in China. Data were collected via one-on-one semi-structured interviews and analyzed using directed content analysis, with iterative mapping of codes to COM-B sub-domains.

**Results:**

Fourteen themes were identified across the six COM-B dimensions, comprising eight barriers and six facilitators. Capability barriers included post-stroke physical exhaustion and low digital confidence, while facilitators were perceived functional recovery and movement self-correction. Opportunity barriers encompassed physical space limitations, unstable internet connectivity, and social isolation; facilitators were family support and therapist supervision. Motivational barriers included efficacy skepticism, digital fatigue, and re-injury anxiety; facilitators were return-to-work aspiration and routine integration. Adherence was shaped by dynamic interactions among these factors rather than isolated determinants.

**Conclusion:**

Telerehabilitation adherence in young and middle-aged stroke survivors is influenced by a complex interplay of physical, cognitive, environmental, social, and motivational factors. Multi-component interventions that simultaneously target capability, opportunity, and motivation—such as fatigue management, digital skills training, home environment optimisation, family involvement, vocational goal-setting, serious games, and peer support—are warranted to enhance engagement and improve long-term outcomes. The COM-B model provides a useful framework for designing such tailored strategies.

## 1 Introduction

Stroke has emerged as a major global health challenge, representing the leading cause of long-term disability worldwide (1). While traditionally perceived as a disease of the elderly, recent epidemiological data indicate a concerning shift: the incidence of stroke among young and middle-aged adults (aged 18–60 years) has increased significantly over the past two decades (2). In this study, we define young and middle-aged stroke survivors as adults aged 18–60 years, consistent with the age range used in recent Chinese epidemiological and rehabilitation research on working-age stroke populations (3). For this demographic, stroke occurs during their most productive years, leading to profound socio-economic consequences, including loss of employment, long-term psychological distress, and a diminished quality of life (4). Consequently, effective and sustained rehabilitation is crucial to facilitate functional recovery and successful reintegration into society and the workplace.

Post-stroke rehabilitation is a long-term process that requires high intensity and frequency to promote neuroplasticity (5). However, traditional center-based rehabilitation often faces barriers such as high costs, transportation difficulties, and limited medical resources, which are further exacerbated for young and middle-aged patients who must balance recovery with vocational and familial responsibilities (6). Telerehabilitation (TR)—the delivery of rehabilitation services via information and communication technologies—has emerged as a promising alternative, offering flexibility and accessibility (7, 8). Despite its potential to bridge the gap in clinical care, the effectiveness of TR is heavily contingent on patient adherence. Evidence suggests that adherence to home-based TR programs remains suboptimal, with many patients failing to complete the prescribed exercises, thereby compromising clinical outcomes (9, 10).

Adherence is a complex, multi-dimensional behavior influenced by various internal and external factors. Previous studies have identified general barriers to TR, such as technical issues and lack of professional supervision (11). However, young and middle-aged stroke survivors possess unique characteristics, including higher technological literacy, higher recovery expectations, and different life-stage stressors compared to older adults (12). There is a scarcity of qualitative research that deeply explores the specific facilitators and barriers to TR adherence in this particular age group. To design effective interventions, it is imperative to move beyond a descriptive analysis of barriers and adopt a theoretically grounded approach to understand the underlying mechanisms of behavioral change (Figure 1).

**Figure 1:**
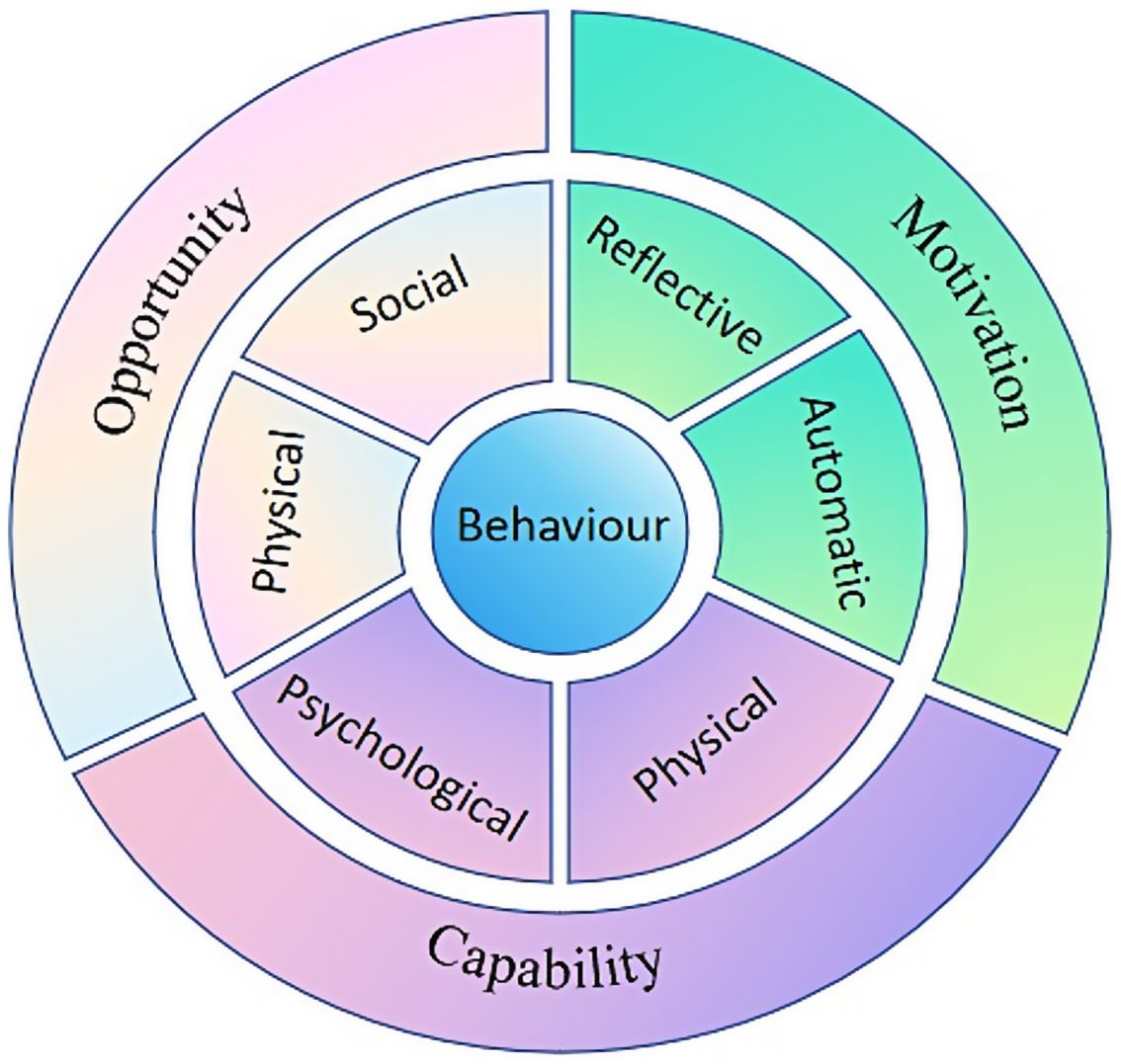
The COM-B framework (Capability, Opportunity, Motivation-Behavior) illustrating the three core components and their sub-domains that influence behavior. This framework was used as the theoretical lens for coding and organizing barriers and facilitators to telerehabilitation adherence.

The Capability, Opportunity, Motivation, and Behavior (COM-B) model, part of the Behavior Change Wheel (BCW), provides a comprehensive framework for understanding health behaviors (13). According to this model, for a behavior to occur, an individual must have the physical and psychological Capability, the social and physical Opportunity, and the Motivation to perform it. The COM-B model has been effectively employed to unpack health-related behaviors in various chronic conditions, including the determinants of post-stroke physical activity and engagement in community-based rehabilitation (14, 15). By applying the COM-B model, researchers can systematically categorize the factors influencing TR adherence, providing a structured foundation for developing targeted behavioral interventions.

Therefore, this study aims to qualitatively explore the experiences and perceptions of young and middle-aged stroke survivors regarding telerehabilitation. By utilizing the COM-B framework, we seek to identify the key factors influencing their adherence, ultimately providing evidence-based recommendations for optimizing TR programs and improving long-term recovery outcomes for this active population.

## 2 Methods

### 2.1 Study design

A qualitative descriptive design was adopted, underpinned by a pragmatic epistemological stance. This approach was chosen to generate straight, data-near descriptions of phenomena that can directly inform intervention development. The study employed semi-structured individual interviews and a directed content analysis, with the Capability, Opportunity, Motivation-Behavior (COM-B) model serving as the theoretical framework. The COM-B model proposes that behavior is a product of an interaction between three components: psychological and physical capability, social and physical opportunity, and reflective and automatic motivation. This study was reported in accordance with the Standards for Reporting Qualitative Research (SRQR) (16).

### 2.2 Participants and recruitment

This study was conducted in the Department of Rehabilitation of a large, tertiary university hospital in Xiamen, China. Participants were prospectively recruited from January 2026 to March 2026. A purposive, maximum-variation sampling strategy was used to recruit participants who could provide rich, diverse information on telerehabilitation adherence. Sampling was guided by a predefined matrix to ensure variation in age (18–60 years), sex, time since stroke onset (3 months to 5 years), level of motor impairment (mild to moderate hemiparesis), type of telerehabilitation platform (app-based vs. video-conferencing), and adherence level (consistent vs. non-consistent).

Inclusion criteria were: (1) first-ever stroke confirmed by neuroimaging, (2) aged 18 to 60 years at enrollment, (3) at least three months post-stroke, (4) having been prescribed and initiated a home-based telerehabilitation programme for at least four weeks within the preceding six months, (5) possessed sufficient cognitive and communication abilities to participate in interviews (Mini-Mental State Examination score ≥ 24), and (6) provided informed consent. Individuals with severe aphasia, untreated major depression, or other progressive neurological conditions were excluded. Recruitment continued until information power was achieved; that is, data were sufficiently rich and no new substantive codes related to the COM-B domains were generated in two consecutive interviews.

### 2.3 Description of the telerehabilitation intervention

All participants had been prescribed a structured home-based telerehabilitation programme by their treating rehabilitation physician at Zhongshan Hospital of Xiamen University. The programme was delivered through one of two modalities, depending on the patient’s preference, device availability, and clinical assessment at discharge:

- App-based telerehabilitation. Participants used a dedicated rehabilitation application that provided pre-recorded video demonstrations of prescribed exercises, accompanied by audio instructions and animated movement models. The app displayed step-by-step exercise sequences and included a built-in timer and repetition counter. Participants were instructed to follow the on-screen model and perform exercises independently at home.
- Video-conferencing telerehabilitation. Participants joined real-time video sessions with a physiotherapist via a secure video-conferencing platform. During these sessions, the therapist demonstrated exercises, observed the participant’s performance in real time, and provided verbal corrections and encouragement.

An overview of the TR intervention structure and weekly workflow is presented in Supplementary material S1.

### 2.4 Data collection

Data were collected through one-on-one semi-structured interviews. An interview guide was developed *a priori* based on the COM-B framework (Supplementary material S2), with open-ended questions and probes targeting each domain:

1. Capability: “What skills or knowledge helped or hindered your use of the TR system?”
2. Opportunity: “How did your home environment, family support, or work schedule affect your rehabilitation routine?”
3. Motivation: “What kept you engaged—or made you consider stopping—the TR program?”

The guide was pilot-tested with two stroke survivors not included in the main study and refined for clarity and cultural appropriateness. Interviews were conducted by YLH in a private clinical room or via encrypted video conferencing (per participant preference), lasted 45–60 min, were audio-recorded with consent, and supplemented with field notes documenting non-verbal cues and contextual observations. Upon completion of the interview, each participant received a small token of appreciation (worth approximately 20 CNY) for their time and contribution. Audio files were transcribed verbatim within 48 h and cross-checked against recordings for accuracy.

### 2.5 Data analysis

A directed content analysis was conducted, integrating the deductive application of the COM-B model with the inductive generation of subthemes. This hybrid approach was selected because the COM-B model provided a comprehensive, theoretically coherent lens for systematically mapping adherence determinants, while maintaining openness to emergent codes ensured that context-specific nuances unique to young and middle-aged survivors were preserved. The analysis proceeded through six structured steps using NVivo 10.

The analysis process followed six structured steps:

Step 1: Familiarization
  The primary analyst (YLH) and a second coder (MLS) independently read and re-read the first five transcripts to achieve data immersion. Marginal notes were made to identify potential meaning units and initial impressions regarding TR adherence experiences.
Step 2: Generating Initial Codes
  Meaningful text segments related to telerehabilitation adherence were openly coded. Coding captured both manifest (surface-level) and latent (underlying) meanings.
Step 3: Developing a Coding Framework Mapped to COM-B Sub-domains
  The two coders met to compare their open codes and, through consensus, mapped them onto the three *a priori* COM-B domains (Capability, Opportunity, Motivation). Codes that resonated with more than one domain were discussed, and the original interview context was reviewed to reach an agreement. A detailed codebook was constructed, containing each code name, a clear definition, and an anchor example.
Step 4: Applying the Framework
  The finalized codebook was systematically applied to all remaining transcripts by the primary analyst, who also recorded any newly emerging codes that did not fit the existing structure. To ensure consistency, the second coder independently coded a random 20% of the transcripts. Inter-coder reliability was maintained through regular discussion and consensus meetings rather than numerical coefficients, consistent with qualitative interpretivist practices. Discrepancies were resolved through dialogue or consultation with a third senior researcher.
Step 5: Categorizing Barriers and Facilitators within Sub-domains
  Codes within each COM-B domain were iteratively clustered into subcategories and then aggregated into themes that described a barrier or facilitator.
Step 6: Reviewing and Defining Themes
  The full research team reviewed the candidate themes against the coded extracts and full transcripts, refining theme boundaries and ensuring coherence. Any codes that did not fit the COM-B model were examined for a potential new domain or theme; in this study, they informed contextual descriptors rather than an additional theoretical domain. Final themes were named and defined with verbatim quotes selected to illustrate each.

Throughout analysis, the reflexive journal and regular peer debriefing sessions with a senior qualitative researcher not directly involved in data collection were used to challenge emerging interpretations and enhance analytical integrity. An audit trail was maintained, documenting key coding decisions, theme development, and modifications to the analytic framework. Saturation was considered achieved after 12 interviews, when no new subthemes or relevant properties emerged. Three additional interviews were conducted, confirming saturation.

### 2.6 Techniques to enhance trustworthiness

Several strategies aligned with Lincoln and Guba’s (17) trustworthiness criteria were employed. Credibility was supported by investigator triangulation (multidisciplinary team), an audit trail documenting all analytical decisions, and member checking: a plain-language summary of the emerging themes was sent to five participants, and three responded with comments that affirmed or clarified our interpretations, which were subsequently integrated. Transferability was addressed through thick description of the participants, context, and processes, enabling readers to assess the applicability of findings to their own settings. Dependability and confirmability were enhanced by maintaining a comprehensive audit trail of raw data, transcripts, field notes, codebook iterations, and reflexive journals, and by conducting an external inquiry audit with a qualitative methodologist not affiliated with the project. The team also openly acknowledged the influence of the COM-B framework as both a lens and a potential blindfold and actively sought disconfirming evidence during analysis.

### 2.7 Ethical considerations

Ethical approval was obtained from the Institutional Review Board of Zhongshan hospital, Xiamen University (Approval No. 2025-011). Written informed consent was secured prior to data collection. Participants were informed of their right to withdraw at any time without affecting clinical care. All data were anonymized using pseudonyms; audio files and transcripts were stored on password-protected institutional servers accessible only to the research team. Findings are reported in aggregate with de-identified quotations to protect participant confidentiality. The study adhered to the Declaration of Helsinki.

## 3 Results

### 3.1 Participant characteristics

Between January 2026 to March 2026, 15 young and middle-aged stroke survivors (9 male, 6 female; median age 48 years, range 32–58) completed the interview. The median time post-stroke was 11 months (range 4–38). All participants had been prescribed and had initiated a home-based telerehabilitation programme for a minimum of four weeks. Adherence was operationally defined by average weekly exercise days extracted from the platform usage log over the initial 4-week intervention period. Nine participants (60%) were classified as adherent (≥5 days/week), while six (40%) were non-adherent (<5 days/week). Other demographic and clinical characteristics are presented in Table 1.

**Table 1:** Participant characteristics (*N* = 15).

ID	Age (years)	Sex	Stroke type	Time post-stroke (months)	Motor impairment	Telerehab platform	Adherence level
P01	45	Male	Ischaemic	8	Mild	App-based	Adherent
P02	52	Female	Haemorrhagic	15	Moderate	Video-conferencing	Non-adherent
P03	38	Male	Ischaemic	6	Mild	App-based	Adherent
P04	48	Female	Ischaemic	22	Mild	Video-conferencing	Adherent
P05	55	Male	Haemorrhagic	38	Moderate	App-based	Non-adherent
P06	32	Male	Ischaemic	4	Mild	Video-conferencing	Adherent
P07	58	Female	Ischaemic	12	Moderate	App-based	Non-adherent
P08	41	Male	Haemorrhagic	9	Moderate	Video-conferencing	Adherent
P09	50	Female	Ischaemic	18	Mild	App-based	Non-adherent
P10	36	Male	Ischaemic	5	Mild	App-based	Adherent
P11	47	Male	Ischaemic	30	Moderate	Video-conferencing	Non-adherent
P12	54	Female	Haemorrhagic	14	Mild	App-based	Adherent
P13	43	Male	Ischaemic	10	Mild	Video-conferencing	Adherent
P14	57	Male	Ischaemic	26	Moderate	App-based	Non-adherent
P15	49	Female	Ischaemic	7	Mild	App-based	Adherent

Adherence group was determined by average weekly exercise days over the initial 4-week programme. Adherent = ≥5 days/week; Non-adherent = <5 days/week. Motor impairment: mild = Fugl-Meyer Assessment upper-extremity subscore ≥50; moderate = 30–49.

### 3.2 Overview of findings

Directed content analysis identified 14 themes mapped onto the six dimensions of the COM-B model, comprising eight barriers and six facilitators. The barriers were post-stroke physical exhaustion, low digital confidence, physical space limitations, unstable internet connectivity, social isolation, efficacy skepticism, digital fatigue, and re-injury anxiety. The facilitators were perceived functional recovery, movement self-correction, family support, therapist supervision, return-to-work aspiration, and routine integration. No substantial thematic differences were observed between ischaemic and haemorrhagic participants, though the small number of haemorrhagic cases limited definitive inference. Core themes were similar across platform types, yet video-conferencing users more frequently cited therapist supervision as a facilitator, whereas app-based users more often reported digital fatigue and low digital confidence (Table 2).

**Table 2:** Themes influencing telerehabilitation adherence among young and middle-aged stroke survivors mapped to the COM-B model.

COM-B component	Sub-domain	Theme	Type
Capability	Physical Capability	1. Post-stroke physical exhaustion	Barrier
		2. Perceived functional recovery	Facilitator
	Psychological Capability	3. Low digital confidence	Barrier
		4. Movement self-correction	Facilitator
Opportunity	Physical Opportunity	5. Physical space limitations	Barrier
		6. Unstable internet connectivity	Barrier
	Social Opportunity	7. Social isolation	Barrier
		8. Family support	Facilitator
		9. Therapist supervision	Facilitator
Motivation	Reflective Motivation	10. Efficacy skepticism	Barrier
		11. Return-to-work aspiration	Facilitator
	Automatic Motivation	12. Digital fatigue	Barrier
		13. Routine integration	Facilitator
		14. Re-injury anxiety	Barrier

#### 3.2.1 Capability

##### 3.2.1.1 Physical capability

###### 3.2.1.1.1 Theme 1: post-stroke physical exhaustion (Barrier)

Pathological fatigue and residual motor deficits (e.g., spasticity, incoordination) physically limit the ability to initiate or complete prescribed exercise sessions.

*“I wake up tired, and by exercise time my body feels like a drained battery. I just can’t lift my arm for 20 minutes.” (P14)*

*“My hand curls up and I can’t open it for the grip exercise. It hurts and I end up using the wrong muscles.” (P07)*

###### 3.2.1.1.2 Theme 2: perceived functional recovery (facilitator)

Self-observed improvements in strength, movement quality, or daily activities reinforce belief in the programme and motivate continued participation.

“Last week I couldn’t hold a cup. Now I can grasp it. Seeing that change makes me want to do even more.” *(P03)*

“My leg feels stronger on stairs. That’s my proof the sessions work.” *(P10)*

##### 3.2.1.2 Psychological capability

###### 3.2.1.2.1 Theme 3: low digital confidence (barrier)

Low digital confidence emerged as a significant cognitive barrier. Many participants, particularly those in the older age range, struggled to navigate the telerehabilitation platform independently. This difficulty was compounded by problems recalling multi-step exercise instructions, leading to confusion, fear of error, and ultimately avoidance:

*“I accidentally closed the app and could not find it again. If my son is not home, I just give up.” (P09)*

*“She showed me five exercises. When I’m alone, I only remember the first two. I’m scared of doing the wrong thing.” (P07)*

###### 3.2.1.2.2 Theme 4: movement self-correction (facilitator)

The ability to perform movement self-correction was a key facilitator of safe and effective independent practice. Participants who could visually or proprioceptively compare their own movements with the on-screen model and make real-time adjustments reported greater confidence and autonomy:

*“The video is my mirror. I watch my arm and compare it to the screen. If I’m off, I pause and re-align.” (P13)*

*“I can feel when my shoulder hikes up, so I slow down. The therapist taught me that self-check.” (P06)*

#### 3.2.2 Opportunity

##### 3.2.2.1 Physical opportunity

###### 3.2.2.1.1 Theme 5: physical space limitations (barrier)

Insufficient physical space at home was a common environmental barrier. Participants described narrow living areas that required significant rearrangement of furniture before each session, which created friction and discouraged regular practice:

*“Our living room is narrow. I have to move the sofa and coffee table every time.” (P09)*

###### 3.2.2.1.2 Theme 6: unstable internet connectivity (barrier)

Unstable internet connectivity constituted a distinct technological barrier. Frequent video freezing, audio lag, and unexpected disconnections disrupted the flow of telerehabilitation sessions, leading to frustration and diminished motivation:

*“The video freezes and the audio lags. I wait and wait, and then my motivation is gone.” (P05)*

##### 3.2.2.2 Social opportunity

###### 3.2.2.2.1 Theme 7: social isolation (barrier)

Social isolation was a prominent psychosocial barrier. Rehabilitating alone at home without peer interaction, encouragement, or social benchmarking fostered loneliness and diminished motivation. Participants missed the collective energy of in-person group settings:

*“In the hospital we encouraged each other. At home, it’s dead silent. I miss that push.” (P07)*

*“I’m the only one in my circle with a stroke. If I saw others like me doing it, maybe I’d push harder.” (P11)*

###### 3.2.2.2.2 Theme 8: family support (facilitator)

Family support emerged as a critical interpersonal facilitator. Cohabiting family members, particularly spouses and adult children, provided practical hands-on assistance with device setup and physical guarding during exercises. Their verbal encouragement and daily reminders created a supportive micro-environment that reduced barriers and fostered adherence:

*“My husband puts the tablet on the stand and holds my waist during balance training. Without him, I would not dare do it.” (P04)*

*“My daughter sets up the tablet every evening and stays nearby until I finish. Her presence keeps me accountable.” (P01)*

###### 3.2.2.2.3 Theme 9: therapist supervision (facilitator)

Therapist supervision, delivered through remote check-ins and personalized feedback, served as a distinct facilitator. The sense of being professionally monitored—even at a distance—cultivated a feeling of accountability. Participants expressed a desire not to disappoint their therapist and valued the ongoing therapeutic connection:

*“Every Friday my therapist sends a voice message asking how my week went. Knowing she reviews my data makes me feel I owe her my effort.” (P15)*

*“He checks my uploaded videos and gives me corrections. I feel like I’m still under his care, not abandoned.” (P01)*

#### 3.2.3 Motivation

##### 3.2.3.1 Reflective motivation

###### 3.2.3.1.1 Theme 10: efficacy skepticism (barrier)

Doubts regarding the clinical equivalence of remote delivery compared to in-person therapy, or perceived misalignment between prescribed exercises and individual recovery trajectories, led to gradual disengagement.

*“Screen exercises cannot replace hands-on manual therapy. When progress plateaus, I question whether continuing is actually worthwhile.” (P11)*

*“At first I doubted it—just a screen? How could that possibly help?” (P02)*

###### 3.2.3.1.2 Theme 11: return-to-work aspiration (facilitator)

Clear, future-oriented goals centered on vocational reintegration drove sustained self-monitoring. Participants who explicitly linked daily exercise completion to career milestones demonstrated stronger behavioral persistence.

*“I want to return to my teaching job. Every squat is a step back to the podium.” (P01)*

*“I log every session because I must pass the occupational health assessment by October.” (P04)*

##### 3.2.3.2 Automatic motivation

###### 3.2.3.2.1 Theme 12: digital fatigue (barrier)

Digital fatigue emerged as a distinct automatic barrier. The repetitive and sensorially unchanging nature of the app-based content led to cumulative boredom and psychological exhaustion. Participants described a negative emotional response that made them avoid opening the platform:

*“Same music, same robot voice, same exercises day after day. I got so bored I could not bear to open it.” (P02)*

*“It feels like a chore, not therapy. My mood drops the moment I see the loading screen.” (P09)*

###### 3.2.3.2.2 Theme 13: routine integration (facilitator)

Embedding telerehabilitation into established daily rituals reduced reliance on conscious willpower over time, transitioning adherence from effortful compliance to automatic habit.

*“It started as a chore, but now it flows naturally after my morning coffee. I no longer need to convince myself to start.” (P15)*

*“It’s like brushing my teeth. After breakfast, my feet just walk me to the corner. I do not even think about it.” (P08)*

###### 3.2.3.2.3 Theme 14: re-injury anxiety (barrier)

Re-injury anxiety was identified as an emotional barrier that specifically undermined unsupervised exercise engagement. A heightened fear of falling, overstraining, or triggering another stroke during home-based practice led participants to avoid or prematurely terminate sessions. This fear was particularly pronounced among those who lacked immediate access to professional supervision:

*“I’m worried about falling” (P07)*

*“Every time I stand up for the balance exercise, I think, ‘What if I fall and hit my head again?’ That fear freezes me.” (P14)*

### 3.3 Interaction between COM-B components

Adherence was not determined by isolated factors but emerged from dynamic interactions across the COM-B system. Return-to-work aspiration, though a powerful reflective driver, did not guarantee adherence when post-stroke physical exhaustion and physical space limitations coexisted. In such cases, participants described a frustrating gap between their vocational goals and their bodily or environmental capacity to act on them. Re-injury anxiety often intensified in the absence of therapist supervision, particularly among participants with unstable internet connectivity that prevented real-time guidance during challenging exercises. This fear-avoidance cycle further reduced engagement and reinforced perceptions of physical vulnerability. Conversely, robust family support frequently compensated for digital fatigue and re-injury anxiety by providing external accountability and a reassuring physical presence. Similarly, routine integration served as a protective factor that buffered against the motivational erosion caused by efficacy skepticism. These interdependencies underscore the necessity of multi-component interventions that simultaneously address capability, opportunity, and motivation to optimize telerehabilitation adherence in this population.

## 4 Discussion

This qualitative study used the COM-B model to systematically explore the barriers and facilitators to telerehabilitation adherence among young and middle-aged stroke survivors. Fourteen themes were identified across the six COM-B dimensions, comprising eight barriers and six facilitators. These findings extend the existing literature by providing a theoretically grounded, multi-level account of adherence behavior in a population that faces unique challenges at the intersection of stroke recovery, midlife responsibilities, and digital health engagement.

Post-stroke physical exhaustion emerged as a pervasive barrier, consistent with evidence that pathological fatigue affects up to 48% of stroke survivors and independently predicts reduced physical activity participation (18). In the current study, fatigue was frequently described in conjunction with residual motor deficits such as spasticity, creating a compound barrier that undermined exercise initiation and completion. This finding aligns with quantitative research demonstrating that baseline fatigue severity is negatively associated with telerehabilitation session attendance (6). Conversely, perceived functional recovery served as a key facilitator. Participants who observed tangible improvements in strength or daily function reported enhanced self-efficacy, which in turn motivated continued engagement. This is consistent with social cognitive theory and empirical work showing that mastery experiences are among the strongest predictors of exercise adherence in neurological populations (19). From a clinical perspective, telerehabilitation platforms should incorporate objective progress-tracking features to make incremental gains visible and reinforce self-efficacy (20).

Low digital confidence was a prominent psychological barrier, particularly among participants in the older end of the study age range. Difficulties navigating the platform interface and recalling multi-step exercise sequences led to confusion and avoidance. This finding resonates with the digital health literacy literature, which identifies low eHealth literacy as a significant predictor of non-usage and non-adherence in technology-delivered interventions (21, 22). The cognitive load imposed by post-stroke cognitive changes likely compounds this challenge, as executive function and working memory deficits are common even among individuals with good overall functional recovery (23). In contrast, movement self-correction ability facilitated safe independent practice. This skill reflects embodied self-awareness and can be cultivated through explicit training strategies such as video-based self-modeling and therapist-guided error detection exercises (24).

Environmental constraints, specifically physical space limitations and unstable internet connectivity, represented distinct barriers. These findings extend prior work identifying inadequate home environment as a barrier to home-based rehabilitation (25) by disentangling spatial and technological dimensions. Participants living in compact urban apartments described the burdensome preparation required before each session, while those with poor connectivity experienced frustration and disengagement. These issues are particularly relevant in low- and middle-income contexts where housing conditions and digital infrastructure may be suboptimal (26). Social isolation emerged as a psychosocial barrier, echoing research on the psychological impact of home-based rehabilitation conducted without peer interaction (27). Participants contrasted the silence of home-based practice with the collective energy of in-person group therapy, highlighting the importance of social comparison and mutual encouragement.

Family support and therapist supervision were identified as distinct facilitators within the social opportunity domain. The practical and emotional scaffolding provided by cohabiting family members—device setup, physical guarding, daily reminders—created a microenvironment conducive to adherence. This finding is consistent with the chronic illness literature, where family involvement has been shown to improve treatment adherence through both instrumental and emotional pathways (28, 29). Therapist supervision, delivered via remote check-ins and personalized feedback, fostered a sense of accountability and ongoing therapeutic connection. Previous studies have emphasized the importance of the therapeutic alliance in telemedicine contexts, noting that regular, brief contact can mitigate the perceived distance inherent in remote delivery models (30, 31). These findings suggest that hybrid models combining family-mediated support with scheduled therapist contact may optimize adherence outcomes.

Efficacy skepticism was a reflective barrier that led to gradual disengagement. This is consistent with the concept of treatment credibility, which has been identified as a determinant of adherence across various rehabilitation modalities (32). In the current study, skepticism was often expressed by participants who had previously received hands-on manual therapy and perceived a qualitative difference. Addressing such beliefs through patient education about the evidence base for telerehabilitation and through blended care models that combine in-person and remote sessions may be beneficial (7, 33). Return-to-work aspiration, on the other hand, emerged as a powerful facilitator uniquely salient to this young and middle-aged cohort. Participants who explicitly linked daily exercise to vocational milestones demonstrated stronger behavioral persistence. This aligns with the goal-setting literature, which indicates that personally meaningful, specific goals enhance motivation and adherence more effectively than generic health-related goals (34, 35). Incorporating vocational goal-setting into telerehabilitation programmes may therefore be a targeted strategy for enhancing engagement in working-age stroke survivors.

Digital fatigue was identified as a barrier rooted in the repetitive, sensorially unchanging nature of app-based content. Participants described cumulative boredom that eroded their initial motivation over time. This phenomenon has been increasingly recognized in the digital health literature, with studies showing that content variety and interactive features are important determinants of sustained user engagement (36, 37). Recent work has demonstrated that serious rehabilitation games, which embed therapeutic exercises within gamified, interactive environments, can enhance stroke survivors’ willingness to participate in telerehabilitation and improve functional outcomes. By introducing elements such as scoring systems, narrative contexts, and progressively challenging levels, serious games counteract the monotony that underlies digital fatigue and sustain user motivation over extended periods (38). Re-injury anxiety was another emotional barrier, particularly among those exercising without immediate professional supervision. This finding extends the fear-avoidance model, originally developed in chronic pain research (39), to the telerehabilitation context and suggests that safety reassurance strategies and graded exposure protocols warrant integration into remote programmes. Routine integration served as a protective facilitator, with participants who embedded telerehabilitation into established daily rituals transitioning from effortful compliance to automatic habit. This is consistent with habit formation theory, which posits that behavioral repetition in stable contexts promotes automaticity and reduces reliance on conscious motivation (40, 41).

### 4.1 Implications for practice

These findings have several implications for the design and delivery of telerehabilitation services. First, pre-programme assessments should evaluate not only physical function but also digital literacy, home environmental suitability, and connectivity reliability to identify individuals at risk of non-adherence. Second, telerehabilitation platforms should incorporate features that address multiple COM-B domains simultaneously: progress visualisation to enhance perceived recovery (capability), flexible scheduling and offline functionality to accommodate environmental constraints (opportunity), varied content to combat digital fatigue (automatic motivation), and personalized goal-setting modules targeting vocational outcomes (reflective motivation). Third, hybrid care models that combine periodic in-person sessions with remote delivery may address efficacy skepticism and build therapeutic alliance while preserving the convenience of home-based practice. Fourth, involving family members as co-facilitators and providing them with basic training in exercise supervision and safety monitoring could amplify the benefits of social opportunity. Fifth, incorporating online peer co-rehabilitation communities or virtual group-based sessions could address the social isolation barrier identified in this study. Such platforms enable participants to share experiences, exchange encouragement, and engage in social comparison, recreating the collective motivation that many missed from in-person rehabilitation settings and potentially enhancing long-term adherence.

### 4.2 Strengths and limitations

This study has several strengths, including the use of a well-established theoretical framework, maximum-variation sampling, and the inclusion of both adherent and non-adherent participants. The COM-B model provided a comprehensive lens for mapping the multi-level determinants of adherence, yielding findings that can directly inform intervention development using the Behavior Change Wheel. However, several limitations should be acknowledged. First, the study was conducted at a single tertiary hospital in China, which may limit transferability to settings with different healthcare systems, rehabilitation models, or digital infrastructures. Second, adherence was assessed using objective platform-derived usage logs, which reliably capture exercise frequency but do not reflect the quality, intensity, or correct execution of the exercises performed. Third, the sample comprised participants who had completed at least four weeks of telerehabilitation; individuals who discontinued earlier may have experienced different or more severe barriers that were not captured. Additionally, the small number of participants with haemorrhagic stroke (n = 4) limited our ability to explore potential aetiology-related differences in adherence determinants. Fourth, as a qualitative descriptive study, the COM-B model served as a theoretical framework for deductive thematic analysis rather than a quantitative measurement instrument. Accordingly, no numerical COM-B scores were generated, and statistical associations between demographic variables and COM-B dimensions fall outside the scope of this design. Finally, although member checking was performed with a subset of participants, the findings represent a co-construction between participants and researchers, and alternative interpretations remain possible.

## 5 Conclusion

This qualitative study identified fourteen distinct barriers and facilitators to telerehabilitation adherence among young and middle-aged stroke survivors, systematically organized within the COM-B framework. Key barriers included post-stroke physical exhaustion, low digital confidence, physical space limitations, unstable internet connectivity, social isolation, efficacy skepticism, digital fatigue, and re-injury anxiety. Facilitators encompassed perceived functional recovery, movement self-correction, family support, therapist supervision, return-to-work aspiration, and routine integration. The findings underscore that adherence is not determined by a single factor but emerges from dynamic interactions among capability, opportunity, and motivation. Clinically, these results highlight the need for multi-component interventions that integrate progress tracking, digital literacy support, environmental optimisation, family involvement, therapist accountability, vocational goal-setting, gamified content, peer support, and routine anchoring to maximize engagement. By addressing these multi-level determinants, future telerehabilitation programmes can be better tailored to the unique needs of working-age stroke survivors, ultimately improving functional outcomes and quality of life.

## Data Availability

The original contributions presented in the study are included in the article/Supplementary material, further inquiries can be directed to the corresponding author.

## Supplementary material

SUPPLEMENTARY TABLE S1 Overview of the telerehabilitation intervention structure.

SUPPLEMENTARY TABLE S2 Semi-structured interview guide based on the COM-B framework.
