# Supplementary material for: Barriers and facilitators to telerehabilitation adherence in young and middle-aged stroke survivors: a qualitative COM-B analysis
Source: Front Neurol. 2026 Sep 28;17:1885751. doi: 10.3389/fneur.2026.1885751 (PMC13654838; doi:10.3389/fneur.2026.1885751)
Supplement: Supplementary file 1 [file Table_1.DOCX]

Supplementary material S1

Structure and Weekly Workflow of the Telerehabilitation Intervention
