# Supplementary material for: Barriers and facilitators to telerehabilitation adherence in young and middle-aged stroke survivors: a qualitative COM-B analysis
Source: Front Neurol. 2026 Sep 28;17:1885751. doi: 10.3389/fneur.2026.1885751 (PMC13654838; doi:10.3389/fneur.2026.1885751)
Supplement: Supplementary file 2 [file Table_2.DOCX]

Supplementary material S2. Semi-structured Interview Guide

| COM-B Domain | Anchor Question | Targeted Probes |
| --- | --- | --- |
| Physical Capability | What physical challenges do you experience when starting or completing TR sessions? | - How does post-stroke fatigue or muscle stiffness affect your ability to exercise? - Do residual motor deficits make device handling or movement execution difficult? - How do you manage physical discomfort orpain during unsupervised practice? |
| Psychological Capability | What skills or knowledge helped orhindered your use of the TR system? | - How easy or difficult is it to navigate theapp/interface independently? - Do you struggle to remember multi-stepexercise sequences or interpret progress metrics? - How do you check your own form or correct movements without a therapist present? - What strategies do you use when you feelconfused or unsure during a session? |
| Physical Opportunity | How did your home environment or work schedule affect your rehabilitation routine? | - Do you have a dedicated, quiet space for exercises? If not, what adjustments are needed? - How reliable is your internet connection or device compatibility during live sessions? - Does your work schedule or daily commitments allow flexible timing for TR? - What environmental changes would make practicing at home easier? |
| Social Opportunity | How do other people influence your telerehabilitation routine? | - Who helps you with device setup, safety spotting, or daily reminders? - How do you feel about rehabilitating alone at home compared to group therapy? - How often do you receive feedback or check-ins from your therapist? - Would connecting with other stroke survivors doing TR be helpful? Why or why not? |
| Reflective Motivation | What kept you engaged—or made you consider stopping—the TR program? | - What are your primary goals for doing TR? - How do you track or recognize your own progress? - Have you ever doubted whether remote exercises are as effective as in-clinic therapy? - How do your long-term life goals influence your daily commitment to TR? |
| Automatic Motivation | How do you feel emotionally when it’s time to start a TR session? | - Has TR become a natural part of your daily routine, or does it still require conscious effort? - Do you experience boredom, frustration, or mental fatigue with repetitive app content? - How does fear of falling, re-injury, or overexertion affect your willingness to practice? - What features (e.g., sounds, visuals, progress alerts) make you want to open the platform—or avoid it? |
